# Chemical and Physical Culture Conditions Significantly Influence the Cell Mass and Docosahexaenoic Acid Content of *Aurantiochytrium limacinum* Strain PKU#SW8

**DOI:** 10.3390/md19120671

**Published:** 2021-11-26

**Authors:** Xiaohong Chen, Biswarup Sen, Sai Zhang, Mohan Bai, Yaodong He, Guangyi Wang

**Affiliations:** 1 Center of Marine Environmental Ecology, School of Environmental Science and Engineering, Tianjin University, Tianjin 300072, China; xh_chen2011@tju.edu.cn (X.C.); bsen@tju.edu.cn (B.S.); yaodong.he@tju.edu.cn (Y.H.); 2 Polar Research Institute of China, Shanghai 200136, China; zhangsai@tju.edu.cn; 3 College of Life Sciences, Zhejiang University, Hangzhou 310058, China; bmh@zju.edu.cn; 4 Center for Biosafety Research and Strategy, Tianjin University, Tianjin 300072, China; 5 Key Laboratory of Systems Bioengineering (Ministry of Education), Tianjin University, Tianjin 300072, China

**Keywords:** thraustochytrids, fatty acids, docosahexaenoic acid, cell mass, monosaccharides, salinity

## Abstract

Thraustochytrids are well-known unicellular heterotrophic marine protists because of their promising ability to accumulate docosahexaenoic acid (DHA). However, the implications of their unique genomic and metabolic features on DHA production remain poorly understood. Here, the effects of chemical and physical culture conditions on the cell mass and DHA production were investigated for a unique thraustochytrid strain, PKU#SW8, isolated from the seawater of Pearl River Estuary. All the tested fermentation parameters showed a significant influence on the cell mass and concentration and yield of DHA. The addition of monosaccharides (fructose, mannose, glucose, or galactose) or glycerol to the culture medium yielded much higher cell mass and DHA concentrations than that of disaccharides and starch. Similarly, organic nitrogen sources (peptone, yeast extract, tryptone, and sodium glutamate) proved to be beneficial in achieving a higher cell mass and DHA concentration. PKU#SW8 was found to grow and accumulate a considerable amount of DHA over wide ranges of KH_2_PO_4_ (0.125–1.0 g/L), salinity (0–140% seawater), pH (3–9), temperature (16–36 °C), and agitation (140–230 rpm). With the optimal culture conditions (glycerol, 20 g/L; peptone, 2.5 g/L; 80% seawater; pH 4.0; 28 °C; and 200 rpm) determined based on the shake-flask experiments, the cell mass and concentration and yield of DHA were improved up to 7.5 ± 0.05 g/L, 2.14 ± 0.03 g/L, and 282.9 ± 3.0 mg/g, respectively, on a 5-L scale fermentation. This study provides valuable information about the fermentation conditions of the PKU#SW8 strain and its unique physiological features, which could be beneficial for strain development and large-scale DHA production.

## 1. Introduction

Thraustochytrids are marine unicellular heterotrophic protists that have attracted considerable attention from researchers due to their biotechnological significance in the production of docosahexaenoic acid (DHA, 22:6) and terpenoids [1]. DHA, an omega-3 polyunsaturated fatty acid (PUFA), plays an important role in the brain in terms of visual and neural development and immunity in humans, and therefore has high commercial value [2]. As one of the potential alternative sources of DHA, certain thraustochytrid genera, namely *Aurantiochytrium* and *Schizochytrium*, have shown promising DHA production on an industrial scale. Furthermore, the major advantages of the bioprocess for DHA production by thraustochytrids include the utilization of low-cost substrates, sustainability, possibilities of culture optimization, genetic manipulations, and scaling up [3].

Although a large number of thraustochytrids, isolated from a range of marine habitats, have been reported recently [4,5,6,7,8], the culture conditions of only some of these isolates have been optimized so far for their biotechnological potential. Indeed, to improve the cell mass and DHA content, the optimization of culture conditions is imperative. In particular, the chemical (carbon, nitrogen, salts, potassium, and pH) and physical (temperature, agitation, and dissolved oxygen) culture conditions have a strong bearing on DHA biosynthesis in thraustochytrids [9]. Moreover, because genetic manipulation is cumbersome and may pose public health risks [10], efforts towards the optimization of fermentation conditions are desirable.

Furthermore, previous studies have suggested that the responses of thraustochytrid strains/isolates to culture conditions can be inconsistent [11], and every strain has a unique metabolic potential [2]. In our recent study, the thraustochytrid strain PKU#SW8 was found to possess a novel PUFA synthase subunit B (*pfa*B gene), identified from its genome [12]. The functionality of this subunit was confirmed in a heterologous host and the carbon and nitrogen sources were found to differentially influence the expression of the *pfa*B gene and DHA production in PKU#SW8 culture. Furthermore, the pathways for fatty acids or polyketides in PKU#SW8 are incompletely known, suggesting the existence of an unknown but novel mechanism for DHA production [13]. Altogether, these results revealed the unique genomic and metabolic characteristics of PKU#SW8, including its adaptability to different environmental conditions, regulation of *pfa*B, and DHA accumulation. Moreover, with the availability of its high-quality genome map [13], this strain holds the unique advantages of being developed into a model thraustochytrid strain for DHA production.

In this study, we have elucidated the unique characteristics of PKU#SW8 from a biotechnological perspective and determined the effects of various chemical and physical culture conditions on its DHA production. Through a one-factor-at-a-time (OFAT) experimental design, this study demonstrates how culture conditions can influence the cell mass and DHA production of PKU#SW8. In addition, the optimal conditions determined from the flask experiments were further validated on a 5-L scale fermenter. This study aims to provide valuable information about the fermentation conditions of strain PKU#SW8 for the development of optimization strategies towards the improvement of DHA production on a larger scale.

## 2. Results

### 2.1. Growth Characteristics and Fatty Acid Composition

**Figure 1:**
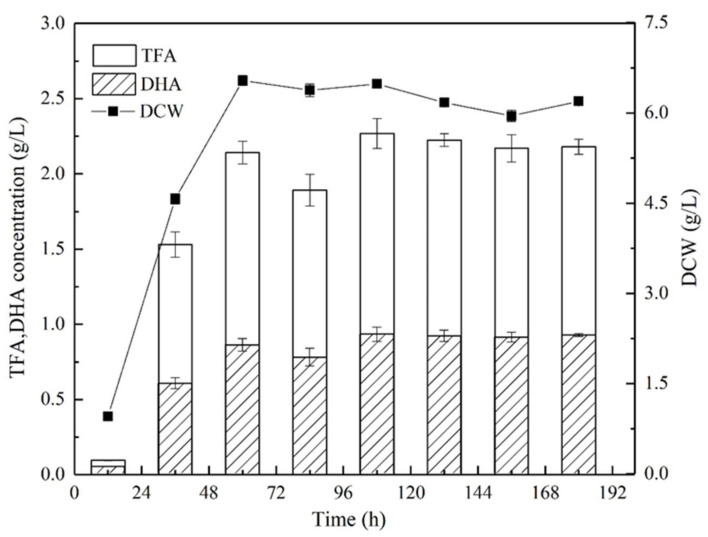
Time course of DCW and concentrations of TFA and DHA with basic (M4) medium in flask culture. The data are expressed as mean ± SD of triplicate experiments.

The stationary phase of growth was observed after 60 h when the PKU#SW8 strain was grown in M4 medium at 28 °C with an agitation speed of 170 rpm (Figure 1). The culture started to accumulate lipids in the exponential phase and showed the maximum accumulation in the stationary phase. The maximum DHA concentration (0.84 ± 0.05 g/L) and total fatty acids (TFA) concentration (2.10 ± 0.13 g/L) were noted at 108 h of growth. The intracellular fatty acids that constituted ≥1% of the TFA were myristic acid (C14:0), pentadecanoic acid (C15:0), palmitic acid (C16:0), eicosapentaenoic acid (C20:5, n-3), docosapentaenoic acid (C22:5, n-6), and DHA (C22:6, n-3) (Table 1). Among these fatty acids, DHA and palmitic acid constituted 40% and 39.7% of the TFA, respectively.

### 2.2. Effects of Carbon Source

**Figure 2:**
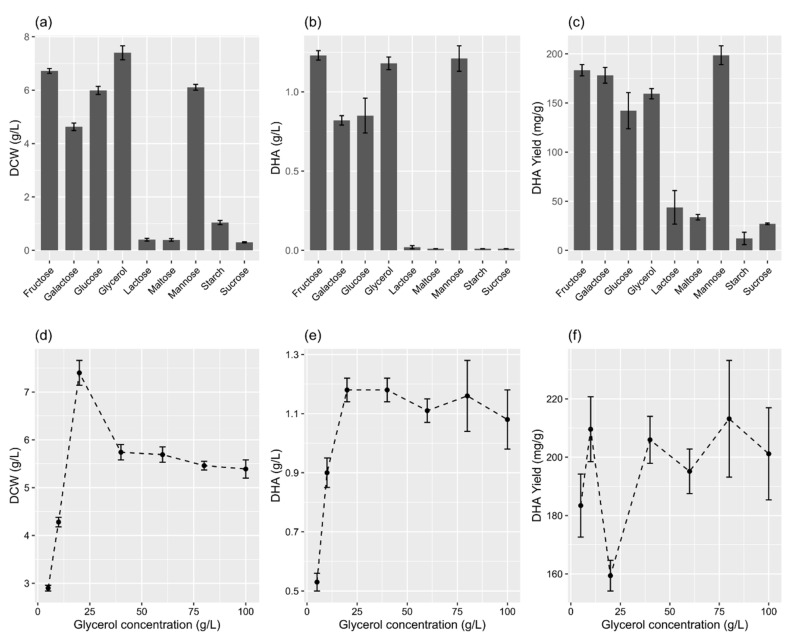
Effects of various carbon sources (**a**–**c**) and glycerol concentrations (**d**–**f**) on DCW and the concentration and yield of DHA in the flask culture. (**a**–**c**) The concentration of individual carbon sources was 20 g/L. The data are expressed as mean ± SD of triplicate experiments.

The means of dry cell weight (DCW) (g/L, F = 8156, *p* < 0.001), DHA concentration (g/L, F = 3276.8, *p* < 0.001), and DHA yield (mg/g, F = 1768.8, *p* < 0.001) for the nine carbon sources were significantly different. Glycerol and the monosaccharides, namely fructose, mannose, glucose, and galactose, were better carbon sources for achieving a good amount of DCW and DHA compared with the disaccharides (lactose, maltose, and sucrose) and polysaccharides (starch) (Figure 2a–c). Glycerol yielded the maximum DCW (7.40 ± 0.26 g/L), whereas sucrose yielded the lowest DCW (0.30 ± 0.02 g/L) (Figure 2a). The differences in the means of DCW between glucose and mannose, and lactose and maltose were non-significant (*p* > 0.05, post hoc test).

Fructose and mannose yielded the maximum concentration (1.23 ± 0.03 g/L) (Figure 2b) and yield (Y_(p/x)_: 198.6 ± 9.6 mg/g, Y_(p/s)_: 61.6 ± 1.6 mg/g) (Figure 2c) of DHA, respectively. The differences in the means of the DHA concentration between fructose and glycerol, fructose and mannose, galactose and glucose, and mannose and glycerol were non-significant (*p* > 0.05, post hoc test). Therefore, glycerol was chosen to further study the effect of the carbon source concentration on DHA accumulation. The glycerol concentration showed a significant effect on the DCW (F = 1363.6, *p* < 0.001), DHA concentration (F = 337.1, *p* < 0.001), and DHA yield (F = 52.78, *p* < 0.001). At 20 g/L of glycerol, the DCW (7.4 ± 0.26 g/L) was at the maximum and a further increase in its concentration resulted in a significantly decreased DCW (Figure 2d). The maximum DHA concentration (1.18 ± 0.04 g/L) was obtained at 20–40 g/L of glycerol (Figure 2e), whereas the maximum DHA yield (213.2 ± 20.0 mg/g) was obtained at 80 g/L of glycerol (Figure 2f).

### 2.3. Effects of Nitrogen Source

**Figure 3:**
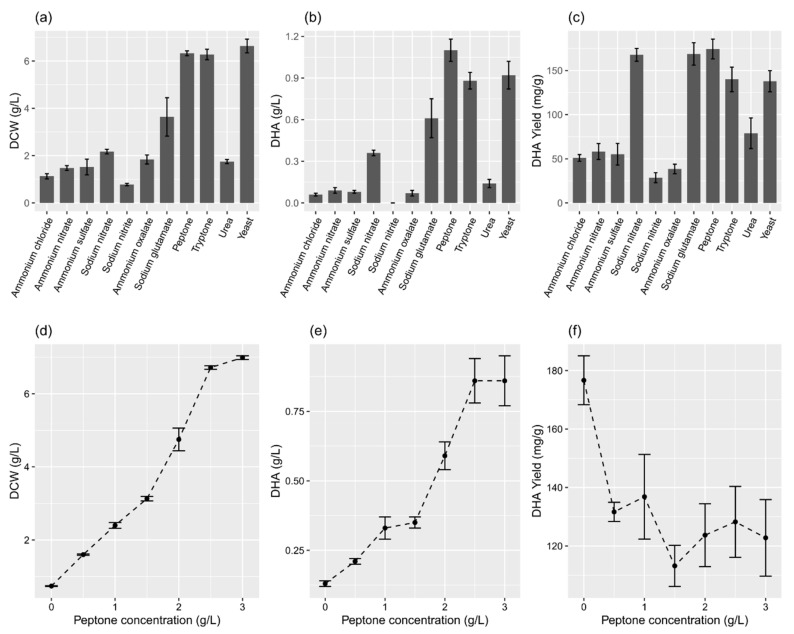
Effects of various nitrogen sources (**a**–**c**) and peptone concentrations (**d**–**f**) on DCW and the concentration and yield of DHA in the flask culture. (**a**–**c**) The concentration of individual nitrogen sources was 2.5 g/L. The data are expressed as mean ± SD of triplicate experiments.

The means of the DCW (F = 1976, *p* < 0.001), DHA concentration (F = 702.8, *p* < 0.001), and DHA yield (F = 339.2, *p* < 0.001) for the 11 nitrogen sources were significantly different. The organic nitrogen sources, except urea, yielded much better DCW and DHA concentrations than the inorganic nitrogen sources (Figure 3a–b). Yeast extract yielded the maximum DCW (6.64 ± 0.29 g/L), whereas sodium nitrite yielded the lowest DCW (0.78 ± 0.05 g/L) (Figure 3a). The differences in the means of DCW between tryptone and peptone, yeast extract and peptone, and tryptone and yeast extract were non-significant (*p* > 0.05, post hoc test). Among the organic nitrogen sources, peptone provided the maximum concentration (1.10 ± 0.08 g/L) (Figure 3b) and yield (174.3 ± 11.2 mg/g) (Figure 3c) of DHA. The differences in the means of the DHA concentration between tryptone and yeast extract were non-significant (*p* > 0.05, post hoc test). Furthermore, the peptone concentration showed a significant effect on the DCW (F = 31935, *p* < 0.001), DHA concentration (F = 346.95, *p* < 0.001), and DHA yield (F = 20.369, *p* < 0.001). The rise in DCW (R^2^ = 0.96, *p* < 0.001) and DHA concentration (R^2^ = 0.88, *p* < 0.001) was fairly associated with the increasing concentration of peptone (Figure 3d–e). The best peptone concentration, which provided the maximum DHA yield (128.2 ± 12.1 mg/g), was 2.5 g/L (Figure 3f).

### 2.4. Effects of KH_2_PO_4_ and Salinity

**Figure 4:**
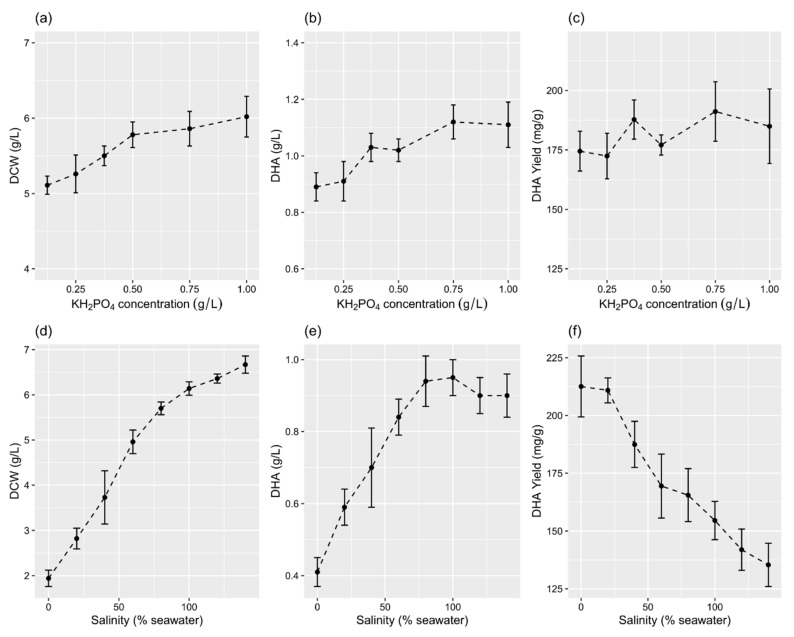
Effects of KH_2_PO_4_ (**a**–**c**) and salinity (**d**–**f**) levels on DCW and the concentration and yield of DHA in flask culture. The data are expressed as mean ± SD of triplicate experiments.

The means of DCW (F = 31.159, *p* < 0.001) and the concentration (F = 21.393, *p* < 0.001) and yield (F = 339.2, *p* < 0.001) of DHA for the various concentrations of KH_2_PO_4_ were significantly different. With an increasing concentration of KH_2_PO_4_, the DCW was generally found to increase (Figure 4a–b). A similar pattern was also noted for the DHA concentration, but only within 0.125–0.75 g/L of KH_2_PO_4_. The maximum yield of DHA (191.1 ± 12.5 mg/g) was achieved at 0.75 g/L of KH_2_PO_4_ (Figure 4c).

The PKU#SW8 strain tolerated a wide range (0–140%) of salinity and accumulated DHA simultaneously. At the same time, the means of DCW (F = 747.66, *p* < 0.001) and the concentration (F = 86.61, *p* < 0.001) and yield (F = 71.67, *p* < 0.001) of DHA for the various levels of salinity were significantly different. Up to 80% seawater, the DCW and DHA concentration increased linearly with the level of salinity (Figure 3d–e). The maximum yield of DHA (212.6 ± 13.2 mg/g) was achieved at 0% seawater (Figure 4f).

### 2.5. Effects of Environmental Variables

**Figure 5:**
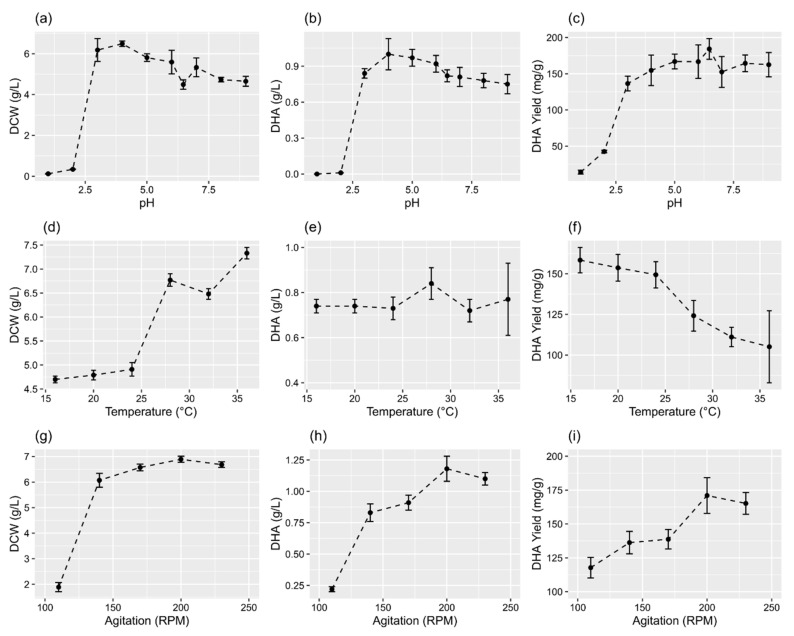
Effects of environmental variables on DCW and the concentration and yield of DHA in flask culture. (**a**–**c**) pH, (**d**–**f**) temperature, and (**g**–**i**) agitation speed. The data are expressed as mean ± SD of triplicate experiments.

The PKU#SW8 strain showed pH tolerance and accumulated a considerable amount of DHA within pH 3–9 (Figure 5a–b). However, the means of DCW (F = 4803.9, *p* < 0.001) and the concentration (F = 1287.1, *p* < 0.001) and yield (F = 461.45, *p* < 0.001) of DHA for the various pH levels were significantly different. The highest DCW (6.49 ± 0.13 g/L) and DHA concentration (1.0 ± 0.13 g/L) were achieved at pH 4.0 (Figure 5a–b), whereas the maximum yield of DHA (184.2 ± 14.3 mg/g) was attained at pH 6.47 (Figure 5c).

The PKU#SW8 strain was able to grow well between 16 °C and 36 °C and accumulate DHA (Figure 5d–e). Although the change in DHA concentration was not remarkable between 16 °C and 36 °C, the increase in the DCW between 24 °C and 28 °C was significantly greater than that between 16 °C and 24 °C. The maximum DCW (7.33 ± 0.12 g/L), concentration (0.84 ± 0.07 g/L) and yield of DHA (158.4 ± 7.8 mg/g) were attained at 36 °C, 28 °C, and 16 °C, respectively (Figure 5d–f).

The DCW (6.07–6.89 g/L) and DHA concentration (0.83–1.18 g/L) were considerable between the agitation speeds of 140 and 230 rpm (Figure 5g–h). Between the agitation speeds of 110 and 200 rpm, the DHA concentration and yield were generally found to increase linearly (Figure 4a–b). The maximum DCW and concentration and yield of DHA were attained at 200 rpm (Figure 5g–i).

### 2.6. DHA Production in a 5-L Fermenter

**Figure 6:**
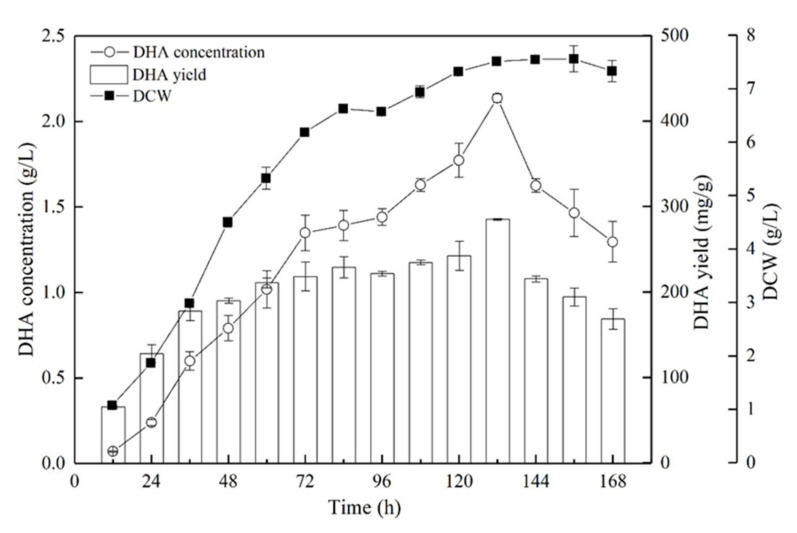
Time course of DCW and the concentration and yield of DHA during fermentation with optimal conditions in a 5-L fermenter. The data are expressed as mean ± SD of triplicate experiments.

With the optimal fermentation conditions (20 g/L glycerol, 2.5 g/L peptone, 80% ASW, pH 4.0, 28 °C, and 200 rpm) determined from the OFAT experiments, the production of DHA was studied in a 5-L fermenter for 168 h (Figure 6). The peak DCW (7.5 ± 0.05 g/L) and DHA concentration (2.14 ± 0.03 g/L) were achieved at 132 h of fermentation. At the same time point, the yield of DHA (282.9 ± 3.0 mg/g) was also the maximum. The concentration of DHA achieved with the optimal fermentation conditions in the 5-L fermenter was 2.5-fold, which was achieved with the basic (M4) medium in flask culture.

## 3. Discussion

### 3.1. Influence of Chemical Culture Conditions

This study provided valuable information for improving the cell mass and DHA accumulation in the PKU#SW8 strain through the evaluation of various culture conditions. The results of testing nine different carbon sources to examine their effects on the cell mass and DHA production revealed that glycerol and monosaccharides such as fructose and mannose are the best substrates for DHA fermentation using PKU#SW8. Previous studies on thraustochytrid isolates have mostly used glucose or glycerol as the preferred carbon sources for DHA production [5,11,14,15,16,17,18,19,20,21]. Our study demonstrated that fructose and mannose are also suitable carbon sources and can yield more than 1 g/L of DHA in PKU#SW8 culture. On the other hand, starch did not prove to be a suitable carbon source for PKU#SW8, which was contrary to a previous report that showed high DCW (14.05 g/L) and a considerable amount of DHA with potato powder [22]. Overall, our study suggests that carbon source utilization patterns can vary markedly among different strains of thraustochytrids, resulting in a wide range of DHA production.

Quantitatively, nitrogen is one of the most important elements contributing to the cell mass of thraustochytrid cells, accounting for up to 2% of cell dry weight [23]. Previous studies have shown that nitrogen sources can significantly affect the DHA accumulation in thraustochytrid strains, such as PKU#SW8 [12], *Aurantiochytrium* sp. KRS101 [16], and *Thraustochytrium* sp. KK17-3 [14]. Higher levels of DHA were obtained when beef extract, corn steep solids, malt extract, or peptone was used as the organic nitrogen source for strain KRS101 [16]. Similarly, peptone and tryptone were the best among the tested organic and inorganic nitrogen sources for strains PKU#SW8 and KK17-3, yielding up to 1.1 g/L [12] and 0.23 g/L [14] of DHA, respectively. In the present study, 11 different nitrogen sources, including six inorganic and five organic sources, were evaluated for their suitability to support DHA production in PKU#SW8 culture (Figure 3). The findings of our study suggest that organic nitrogen sources are much better in improving the DHA production in PKU#SW8 compared with inorganic nitrogen sources, and demonstrated that DHA concentration has a linear relationship with the peptone concentration.

Sodium (as seawater) and potassium (in the form of KH_2_PO_4_) are important factors affecting the growth and fatty acid composition in thraustochytrids [11,24,25,26,27]. In our recent study, we demonstrated that through a sodium-induced mechanism, thraustochytrids shift their energy metabolism from carbohydrate to lipid oxidation for enhanced ATP production [28]. The enhanced ATP production then provides a strong thermodynamic driving force for efficient terpene biosynthesis. Although most thraustochytrid strains show the ability to tolerate a wide range (0–100% seawater) of salinity, the cell mass and fatty acid content are reported to differ among strains [18,22,24,26,29,30,31]. The present study demonstrated that strain PKU#SW8 can grow and accumulate DHA over wide gradients of KH_2_PO_4_ and salinity, between 0.125 and 1.0 g/L and 0 and 140%, respectively. These findings suggest that PKU#SW8 is a potential strain for further investigation towards large-scale DHA production and exploring the role of sodium and potassium in providing a thermodynamic driving force for enhanced DHA biosynthesis.

The pH of the culture medium has profound effects on both the cell growth and fatty acid composition of thraustochytrids [21], perhaps because it is known to affect the absorption of compounds from the culture environment [32]. The experimental results indicated that a pH range of 3–9 is optimal for the growth and DHA production of strain PKU#SW8 (Figure 5a–c). However, although pH between 4.0 and 8.0 has been reported as optimal for the growth of *Schizochytrium* strains, the maximal DHA content can differ among strains [33,34,35,36]. For PKU#SW8, pH 4.0 and pH 6.47 were the best for achieving the maximal content and yield of DHA, respectively. By testing a wide range of pH values, our study revealed that not only a natural pH but a wide pH range (pH 4–9) is suitable for producing DHA in flask culture of PKU#SW8.

### 3.2. Influence of Physical Culture Conditions

The temperature of the culture environment is also one of the important physical conditions influencing the growth and lipid accumulation of thraustochytrids [27,32]. Although thraustochytrid strains are reported to grow over a wide range (15 °C–37 °C) of temperatures [15,18,22,37], temperatures between 25 °C and 30 °C are optimal for biomass production, whereas ~15 °C is optimal for DHA production [27]. For strain PKU#SW8, the biomass production was relatively much higher between 28 °C and 36 °C than between 16 °C and 24 °C, which was in agreement with the previous findings [26]. Furthermore, low temperatures are known to promote the syntheses of DHA and other PUFAs to alter cell membrane fluidity, an adaptation to endure low temperatures [27]. Interestingly, the DHA content of strain PKU#SW8 did not change remarkably between 16 °C and 36 °C, suggesting consistent lipid biosynthesis over a wide temperature range. A similar lipid profile was reported previously for another thraustochytrid strain, PKU#Mn16 [26]. Notably, the unique temperature-dependent lipid profile of PKU#SW8 can be useful in circumventing the need for staged temperature control during fermentation, therefore making it a potential strain for large-scale DHA production.

The agitation speed of the culture flask is known to modulate the mass transfer rates of oxygen, thereby influencing the product yield [38]. Especially for thraustochytrids, oxygen is an essential influencing factor for the synthesis of fatty acids because of the involvement of oxygen-dependent respiratory electron transport and enzymes [39]. In the present study, the biomass production of PKU#SW8 was high at higher agitation speeds (140–230 rpm) and the DHA content was at the maximum between 200 and 230 rpm. These results suggest that a high oxygen mass transfer rate is critical for improving the cell mass and DHA content of PKU#SW8.

## 4. Conclusions

The findings of the present study provide useful information about the chemical and physical culture conditions of strain PKU#SW8 and form the basis for developing strategies towards strain optimization and scaling-up to further improve DHA production. The PKU#SW8 strain, exhibiting typical profiles of salinity, pH, and temperature, seems to be beneficial in large-scale DHA production. Furthermore, PKU#SW8 could serve as a model thraustochytrid strain for investigating the role of various ions (e.g., sodium, potassium, and hydrogen) and temperatures on the bioenergetics of the DHA accumulation process.

## 5. Materials and Methods

### 5.1. Strain and Seed Culture

The thraustochytrid strain PKU#SW8 (CGMCC No. 20069) was previously isolated from seawater in the Delta region of Pearl River, China [8]. The 18S rRNA gene sequence (GenBank accession number: JX847378.1) of this isolate showed 98.7% homology with the 18S rRNA gene sequence of the type strain *Aurantiochytrium limacinum* ATCC MYA-1381. Based on our previous phylogenetic analysis [13] and 18S rRNA full-length sequence identity >97% with *Aurantiochytrium limacinum* ATCC MYA-1381, the strain PKU#SW8 was named the *Aurantiochytrium limacinum* strain PKU#SW8. The genome sequencing data for this strain were deposited in NCBI under BioProject PRJNA419716.

The pure strain was maintained on 2% modified Vishniac’s (MV) [40] agar plates prepared with 100% artificial seawater (ASW, 33 g/L sea salt) at 28 °C and was sub-cultured every four weeks. The MV medium constituted glucose (10 g/L), peptone (1.5 g/L), yeast extract (0.1 g/L), ASW (33 g/L), and agar powder (20 g/L). The artificial seawater was procured commercially (Guangzhou Yier Biological Engineering Co., Ltd., Guangdong, China), which contained Na^+^, Mg^2+^, Ca^2+^, K^+^, Sr^2+^, Cl^−^, SO_4_^2−^, HCO_3_^−^, Br^−^, and other trace minerals such as Cu, Mo, Rb, Be, Co, Ni, I, As, W, Se, Cr, and Mn. The seed culture was prepared by cultivating a single colony from the agar plate in M4 medium (20 g/L glucose, 1.5 g/L peptone, 1.0 g/L yeast extract, 0.25 g/L KH_2_PO_4_, 100% ASW, and pH 7.0) [41] at 28 °C on an orbital shaker at 170 rpm for 36 h.

### 5.2. Batch Experiments

Experiments were performed in shake flasks to evaluate the growth characteristics and DHA accumulation under different culture conditions. The seed culture (5% *w/v*) was transferred to a 100 mL shake flask containing 50 mL of fresh medium. The effects of nine different carbon sources (glucose, glycerol, fructose, sucrose, mannose, galactose, maltose, lactose, and soluble starch) and 11 different nitrogen sources (tryptone, yeast extract, peptone, sodium glutamate, urea, ammonium sulfate, ammonium nitrate, ammonium oxalate, sodium nitrate, and sodium nitrite), and various levels of KH_2_PO_4_ (0.125–1.0 g/L), salinity (0–140%), pH (1–9), temperature (16 °C–32 °C) and agitation speed (110–230 rpm) were investigated. The pH of the culture medium was adjusted to the desired pH with 1 M NaOH or 1 M HCl. In addition, experiments were carried out to study the effects of the various concentrations of the best carbon and nitrogen sources. Except for the variable component, the concentrations of other components of the fermentation medium were the same as that of the M4 medium. All the experiments were performed in triplicate.

With the determined optimal conditions, a batch fermentation was performed in a 5-L fermenter (model: SY-9000-V9, Shanghai Dong Ming Industrial Co. Ltd, Shanghai, China) equipped with DO and pH electrodes, a temperature sensor, impeller, and an air pump. About 300 mL of seed culture, as prepared in Section 5.1, was transferred to the fermentation medium (20 g/L glycerol, 2.5 g/L peptone, 80% ASW, pH 6.5) to bring the final volume to 3 L. The fermentation was carried out at 28 °C with an agitation speed of 200 rpm for 7 days. The dissolved oxygen level was maintained at 30% during fermentation.

### 5.3. Biochemical Analyses

The DCW and DHA were quantified following the methods described in our previous study [26]. Briefly, the cells were harvested by means of centrifugation (Eppendorf 5180R, Eppendorf AG, Germany) at 4000 rpm (2057 *g*) for 10 min, washed twice with sterile distilled water, and then lyophilized for 48 h in a freeze dryer (Christ, Gefriertrocknungsanlagen, Osterode am Harz, Germany). The freeze-dried cells were stored at −80 °C until further processing. The DCW was measured using the gravimetric method. The fatty acid methyl esters were prepared using the direct transesterification method [42] and analyzed following the procedures described in our previous study [43]. The DHA yield, denoted as Y_(p/x)_, is the ratio of DHA (g/L) and DCW (g/L), whereas that denoted as Y_(p/s)_ is the ratio of DHA (g/L) and mannose (g/L). Unless otherwise stated, the DHA yield denotes Y_(p/x)_.

### 5.4. Statistical Analyses

The data are expressed as a mean ± standard deviation (SD). Levene’s test for the homogeneity of variance, the Shapiro–Wilk test of normality, the test of significance (one-way ANOVA), post hoc tests, and regression analysis were performed in R software (version 4.0.0, https://www.r-project.org/). The F statistic is the value obtained from the ANOVA test. The data were plotted using Origin 9.0 software (Origin Lab Corporation, Northampton, MA, USA) and R package ggplot2 (https://ggplot2.tidyverse.org). 

## Figures and Tables

**Table 1:** Major intracellular fatty acid composition of strain PKU#SW8 in basic (M4) medium.

Fatty Acid	Concentration (g/L)	Fraction (%)
C12:0	0.004 ± 0	0.2 ± 0.01
C14:0	0.095 ± 0.006	4.51 ± 0.29
C15:0	0.038 ± 0.002	1.81 ± 0.12
C15:1 (n-5)	0.007 ± 0.001	0.33 ± 0.06
C16:0	0.832 ± 0.056	39.67 ± 2.66
C16:1 (n-7)	0.003 ± 0	0.13 ± 0.02
C17:0	0.01 ± 0.001	0.48 ± 0.03
C18:0	0.02 ± 0.001	0.93 ± 0.06
C20:3 (n-3)	0.009 ± 0.002	0.52 ± 0.03
C21:0	0.011 ± 0.001	0.45 ± 0.09
C20:5 (n-3)	0.057 ± 0.005	2.71 ± 0.24
C22:1 (n-9)	0.009 ± 0.001	0.45 ± 0.04
C22:5 (n-6)	0.152 ± 0.01	7.24 ± 0.46
C22:6 (n-3)	0.839 ± 0.05	40.01 ± 2.4
TFA	2.097 ± 0.127	100
SFA	1.012 ± 0.067	48.25 ± 3.2
PUFA	1.064 ± 0.065	50.74 ± 2.95
MUFA	0.019 ± 0.001	0.89 ± 0.07

Note: TFA, total fatty acids; SFA, saturated fatty acids; PUFA, polyunsaturated fatty acids; MUFA, monounsaturated fatty acids.

## Data Availability

The genome sequencing data for strain PKU#SW8 are available in NCBI BioProject under the PRJNA419716 accession.
